# *N*-*tert*-Butyl-2-methyl­propanamide

**DOI:** 10.1107/S1600536811028947

**Published:** 2011-07-30

**Authors:** Kelly A. Kluge, Diana Fridyland, Cora E. MacBeth, Kenneth I. Hardcastle

**Affiliations:** a Department of Chemistry, Emory University, 1515 Dickey Drive, Atlanta, GA 30322, USA

## Abstract

The title compound, C_8_H_17_NO, crystallizes with two independent mol­ecules in the asymmetric unit. In the crystal, inter­molecular N—H⋯O hydrogen bonding is observed between neighboring mol­ecules, forming continuous mol­ecular chains along the *c*-axis direction.

## Related literature

For the synthesis of the title compound, see: De Kimpe *et al.* (1978 ▶); Christensen *et al.* (1989 ▶); Yasuhara *et al.* (2000 ▶); Li *et al.* (2003 ▶). For its use as a ligand in Zr and Ti complexes, see: Li *et al.* (2003 ▶). For background to the coordination modes of carboxamides, see: Lee & Schafer (2007 ▶).
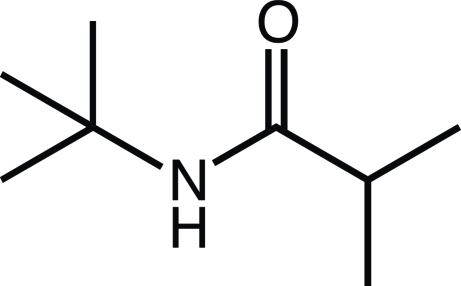


## Experimental

### 

#### Crystal data

C_8_H_17_NO
*M* *_r_* = 143.23
Monoclinic, 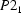

*a* = 9.0378 (6) Å
*b* = 11.3939 (8) Å
*c* = 9.5390 (6) Å
β = 106.133 (3)°
*V* = 943.60 (11) Å^3^
*Z* = 4
Cu *K*α radiation
μ = 0.51 mm^−1^
*T* = 173 K
0.31 × 0.20 × 0.12 mm

#### Data collection

Bruker APEXII CCD diffractometer
Absorption correction: multi-scan (*SADABS*; Bruker, 2008 ▶) *T*_min_ = 0.858, *T*_max_ = 0.941
6358 measured reflections
2662 independent reflections
2624 reflections with *I* > 2σ(*I*)
*R*_int_ = 0.014

#### Refinement

*R*[*F* ^2^ > 2σ(*F* ^2^)] = 0.035
*wR*(*F* ^2^) = 0.106
*S* = 1.00
2662 reflections
181 parameters
1 restraint
H-atom parameters constrained
Δρ_max_ = 0.19 e Å^−3^
Δρ_min_ = −0.16 e Å^−3^
Absolute structure: Flack (1983 ▶), 812 Friedel pairs
Flack parameter: 0.3 (2)

### 

Data collection: *APEX2* (Bruker, 2008 ▶); cell refinement: *SAINT* (Bruker, 2008 ▶); data reduction: *SAINT*; program(s) used to solve structure: *SHELXS97* (Sheldrick, 2008 ▶); program(s) used to refine structure: *SHELXL97* (Sheldrick, 2008 ▶); molecular graphics: *SHELXTL* (Sheldrick, 2008 ▶); software used to prepare material for publication: *SHELXTL*.

## Supplementary Material

Crystal structure: contains datablock(s) global, I. DOI: 10.1107/S1600536811028947/fj2438sup1.cif

Structure factors: contains datablock(s) I. DOI: 10.1107/S1600536811028947/fj2438Isup2.hkl

Supplementary material file. DOI: 10.1107/S1600536811028947/fj2438Isup3.cml

Additional supplementary materials:  crystallographic information; 3D view; checkCIF report

## Figures and Tables

**Table 1:** Hydrogen-bond geometry (Å, °)

*D*—H⋯*A*	*D*—H	H⋯*A*	*D*⋯*A*	*D*—H⋯*A*
N1—H1*A*⋯O2	0.88	2.03	2.8880 (16)	166
N2—H2*A*⋯O1^i^	0.88	2.10	2.9735 (16)	169

Symmetry code: (i) 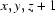.

**Fig. 1.**
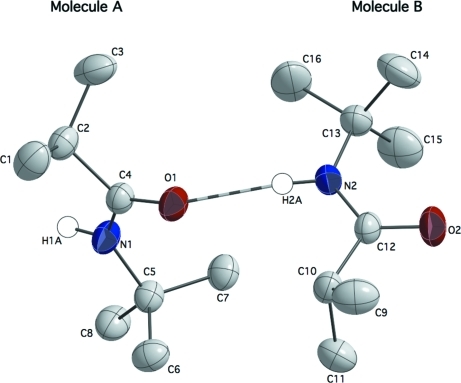
Molecule A and Molecule B of N-tert-butyl-2-methylpropanamide. H atoms except H1A and H2A were omitted for clarity. Thermal ellipsoids are drawn at 50% probability.

**Fig. 2.**
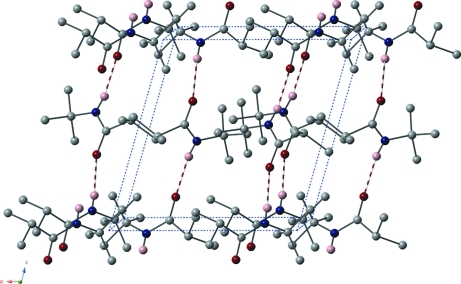
Molecular packing and hydrogen bonding (dashed lines) network of N-tert- butyl-2-methylpropanamide viewed down the b axis.

## supplementary crystallographic information

### Comment

Carboxamides can be deprotonated to form monoanionic amidate ligands. These species can coordinate to transition metal ions through a variety of different coordination modes, including monodentate and bidentate coordination modes, and therefore are coordinatively versatile ligands (Lee & Schafer, 2007). The ease of synthesis of carboxamides make them attractive ligands for a variety of transition metal mediated catalytic reactions, see: Li *et al.* (2003) and Lee & Schafer (2007). Although the synthesis of this compound has been previously described, its solid-state structure has not been reported. The two molecules (A and B) of *N*-*tert*-butyl-2-methylpropanamide (Fig. 1) are stabilized by intermolecular N—H···O hydrogen bonds (Table 1, Fig. 2).

### Experimental

The title molecule was synthesized using a modified literature procedure (Li *et al.*, 2003). Under a nitrogen atmosphere, a 100 ml round bottom flask was charged with 50 ml of dichloromethane, 4.31 ml (41.0 mmol) *tert*-butylamine, 8.55 ml (61.5 mmol) of triethylamine and a stir bar. The solution was cooled to 0 °C and 5.20 ml (49.2 mmol) of isobutyryl chloride was added dropwise. The solution was slowly warmed to room temperature overnight. The resulting pink solution was extracted three times with 50 ml of 0.10 *M* HCl. The organic layer was dried over anhydrous magnesium sulfate, filtered, and concentrated to dryness to yield the desired product in 60% yield. X-ray quality crystals were obtained by slowly evaporating a chloroform solution of the product. The spectroscopic data (NMR, IR, and ESI-MS) match well with the reported values (Li *et al.*, 2003).

### Refinement

The structures were solved using Direct Methods and difference Fourier techniques (*SHELXTL*, V6.12) (Sheldrick, 2008). Hydrogen atoms were added with the HFIX command. These were included in the final cycles of least squares refinement, with isotropic *U^ij^*'s that were determined by the riding model. All non-hydrogen atoms in the main residues were refined anisotropically, but residual solvent molecules in the unit cells were refined isotropically. Structure solution, refinement, and generation of publication materials were performed by using *SHELX*, V6.12 software.

### Crystal data

**Table:**

C_8_H_17_NO	*F*(000) = 320
*M**_r_* = 143.23	*D*_x_ = 1.008 Mg m^−^^3^
Monoclinic, *P*2_1_	Melting point: 393 K
Hall symbol: P 2yb	Cu *K*α radiation, λ = 1.54178 Å
*a* = 9.0378 (6) Å	Cell parameters from 4549 reflections
*b* = 11.3939 (8) Å	θ = 4.8–69.1°
*c* = 9.5390 (6) Å	µ = 0.51 mm^−^^1^
β = 106.133 (3)°	*T* = 173 K
*V* = 943.60 (11) Å^3^	Block, colourless
*Z* = 4	0.31 × 0.20 × 0.12 mm

### Data collection

**Table:**

Bruker APEXII CCD diffractometer	2662 independent reflections
Radiation source: fine-focus sealed tube	2624 reflections with *I* > 2σ(*I*)
graphite	*R*_int_ = 0.014
φ and ω scans	θ_max_ = 69.1°, θ_min_ = 4.8°
Absorption correction: multi-scan (*SADABS*; Bruker, 2008)	*h* = −10→10
*T*_min_ = 0.858, *T*_max_ = 0.941	*k* = −13→12
6358 measured reflections	*l* = −11→11

### Refinement

**Table:**

Refinement on *F*^2^	Secondary atom site location: difference Fourier map
Least-squares matrix: full	Hydrogen site location: inferred from neighbouring sites
*R*[*F*^2^ > 2σ(*F*^2^)] = 0.035	H-atom parameters constrained
*wR*(*F*^2^) = 0.106	*w* = 1/[σ^2^(*F*_o_^2^) + (0.088*P*)^2^ + 0.0504*P*] where *P* = (*F*_o_^2^ + 2*F*_c_^2^)/3
*S* = 1.00	(Δ/σ)_max_ < 0.001
2662 reflections	Δρ_max_ = 0.19 e Å^−^^3^
181 parameters	Δρ_min_ = −0.16 e Å^−^^3^
1 restraint	Absolute structure: Flack (1983), 812 Friedel pairs
Primary atom site location: structure-invariant direct methods	Flack parameter: 0.3 (2)

### Special details

**Table:**

Geometry. All e.s.d.s (except the e.s.d. in the dihedral angle between two l.s. planes) are estimated using the full covariance matrix. The cell e.s.d.'s are taken into account individually in the estimation of e.s.d.'s in distances, angles and torsion angles; correlations between e.s.d.'s in cell parameters are only used when they are defined by crystal symmetry. An approximate (isotropic) treatment of cell e.s.d.'s is used for estimating e.s.d.'s involving l.s. planes.

### Fractional atomic coordinates and isotropic or equivalent isotropic displacement parameters (Å^2^)

**Table:**

	*x*	*y*	*z*	*U*_iso_*/*U*_eq_	
N1	0.30615 (14)	0.07943 (12)	0.55844 (13)	0.0364 (3)	
H1A	0.2841	0.0677	0.6416	0.044*	
N2	0.17705 (14)	0.08317 (12)	1.04274 (12)	0.0355 (3)	
H2A	0.1857	0.1191	1.1263	0.043*	
O1	0.23057 (13)	0.17773 (11)	0.34325 (11)	0.0394 (3)	
O2	0.27727 (15)	0.06614 (13)	0.85203 (12)	0.0498 (3)	
C1	0.1354 (2)	0.35161 (16)	0.5354 (2)	0.0541 (5)	
H1B	0.0593	0.3932	0.5728	0.081*	
H1C	0.1322	0.3819	0.4385	0.081*	
H1D	0.2385	0.3638	0.6018	0.081*	
C2	0.09866 (19)	0.22154 (14)	0.52483 (17)	0.0383 (4)	
H2B	0.1043	0.1912	0.6246	0.046*	
C3	−0.0618 (2)	0.19845 (19)	0.4252 (3)	0.0582 (5)	
H3A	−0.1379	0.2398	0.4627	0.087*	
H3B	−0.0829	0.1140	0.4220	0.087*	
H3C	−0.0680	0.2266	0.3267	0.087*	
C4	0.21920 (17)	0.15640 (14)	0.46654 (15)	0.0342 (3)	
C5	0.43590 (17)	0.01194 (14)	0.53402 (15)	0.0350 (3)	
C6	0.56316 (19)	0.09591 (16)	0.5211 (2)	0.0447 (4)	
H6A	0.5246	0.1463	0.4354	0.067*	
H6B	0.6517	0.0507	0.5105	0.067*	
H6C	0.5948	0.1446	0.6090	0.067*	
C7	0.3832 (2)	−0.06553 (16)	0.39919 (17)	0.0428 (4)	
H7A	0.3015	−0.1183	0.4102	0.064*	
H7B	0.4704	−0.1120	0.3879	0.064*	
H7C	0.3437	−0.0162	0.3127	0.064*	
C8	0.4953 (2)	−0.06704 (17)	0.66758 (18)	0.0466 (4)	
H8A	0.4130	−0.1203	0.6759	0.070*	
H8B	0.5277	−0.0185	0.7556	0.070*	
H8C	0.5832	−0.1129	0.6568	0.070*	
C9	0.4281 (2)	0.28811 (19)	0.9455 (3)	0.0580 (5)	
H9A	0.5106	0.3416	0.9961	0.087*	
H9B	0.3314	0.3318	0.9116	0.087*	
H9C	0.4542	0.2529	0.8617	0.087*	
C10	0.40976 (18)	0.19187 (15)	1.04972 (17)	0.0380 (3)	
H10A	0.3831	0.2280	1.1351	0.046*	
C11	0.5592 (2)	0.12282 (19)	1.1036 (2)	0.0519 (4)	
H11A	0.6423	0.1759	1.1538	0.078*	
H11B	0.5851	0.0865	1.0204	0.078*	
H11C	0.5462	0.0616	1.1714	0.078*	
C12	0.28088 (17)	0.10794 (14)	0.97174 (15)	0.0343 (3)	
C13	0.04849 (19)	−0.00018 (18)	0.99122 (18)	0.0442 (4)	
C14	−0.0612 (2)	0.0436 (2)	0.8479 (2)	0.0677 (6)	
H14A	−0.0073	0.0448	0.7718	0.102*	
H14B	−0.0963	0.1231	0.8618	0.102*	
H14C	−0.1503	−0.0090	0.8183	0.102*	
C15	0.1083 (3)	−0.1214 (2)	0.9736 (3)	0.0666 (6)	
H15A	0.1785	−0.1470	1.0665	0.100*	
H15B	0.1635	−0.1196	0.8985	0.100*	
H15C	0.0216	−0.1762	0.9442	0.100*	
C16	−0.0360 (3)	−0.0012 (3)	1.1101 (3)	0.0796 (8)	
H16B	−0.1251	−0.0539	1.0810	0.119*	
H16C	−0.0710	0.0783	1.1234	0.119*	
H16A	0.0341	−0.0289	1.2020	0.119*	

### Atomic displacement parameters (Å^2^)

**Table:**

	*U*^11^	*U*^22^	*U*^33^	*U*^12^	*U*^13^	*U*^23^
N1	0.0423 (7)	0.0407 (7)	0.0305 (5)	0.0064 (6)	0.0173 (5)	0.0040 (5)
N2	0.0331 (6)	0.0460 (8)	0.0282 (5)	−0.0053 (5)	0.0099 (4)	−0.0068 (5)
O1	0.0445 (5)	0.0478 (7)	0.0295 (5)	0.0060 (5)	0.0159 (4)	0.0053 (5)
O2	0.0592 (7)	0.0643 (8)	0.0309 (5)	−0.0154 (6)	0.0208 (5)	−0.0115 (5)
C1	0.0598 (11)	0.0418 (10)	0.0689 (12)	−0.0012 (8)	0.0318 (9)	−0.0113 (9)
C2	0.0438 (8)	0.0397 (9)	0.0361 (7)	0.0048 (7)	0.0192 (6)	0.0048 (6)
C3	0.0375 (9)	0.0540 (11)	0.0866 (14)	0.0021 (8)	0.0229 (9)	−0.0120 (10)
C4	0.0373 (7)	0.0372 (8)	0.0306 (6)	−0.0009 (6)	0.0135 (5)	−0.0003 (6)
C5	0.0390 (7)	0.0344 (8)	0.0327 (7)	0.0047 (6)	0.0115 (6)	0.0030 (6)
C6	0.0389 (8)	0.0390 (9)	0.0580 (9)	0.0021 (7)	0.0168 (7)	0.0003 (8)
C7	0.0493 (9)	0.0380 (8)	0.0429 (8)	0.0040 (7)	0.0158 (7)	−0.0035 (7)
C8	0.0488 (9)	0.0495 (10)	0.0419 (8)	0.0126 (8)	0.0134 (6)	0.0090 (8)
C9	0.0492 (10)	0.0450 (10)	0.0796 (13)	−0.0033 (8)	0.0175 (9)	0.0128 (10)
C10	0.0388 (7)	0.0406 (8)	0.0378 (7)	−0.0043 (7)	0.0159 (6)	−0.0073 (6)
C11	0.0429 (9)	0.0549 (11)	0.0519 (9)	−0.0034 (8)	0.0031 (7)	−0.0001 (8)
C12	0.0379 (7)	0.0401 (8)	0.0263 (6)	0.0010 (6)	0.0111 (5)	0.0001 (6)
C13	0.0363 (7)	0.0559 (10)	0.0412 (8)	−0.0106 (7)	0.0120 (6)	−0.0078 (8)
C14	0.0457 (10)	0.0811 (15)	0.0627 (12)	−0.0095 (10)	−0.0075 (8)	−0.0083 (11)
C15	0.0647 (13)	0.0491 (11)	0.0838 (15)	−0.0171 (10)	0.0172 (10)	−0.0049 (10)
C16	0.0562 (12)	0.120 (2)	0.0741 (13)	−0.0411 (14)	0.0373 (10)	−0.0240 (15)

### Geometric parameters (Å, °)

**Table:**

N1—C4	1.331 (2)	C7—H7C	0.9800
N1—C5	1.4739 (19)	C8—H8A	0.9800
N1—H1A	0.8800	C8—H8B	0.9800
N2—C12	1.3312 (19)	C8—H8C	0.9800
N2—C13	1.475 (2)	C9—C10	1.520 (3)
N2—H2A	0.8800	C9—H9A	0.9800
O1—C4	1.2328 (18)	C9—H9B	0.9800
O2—C12	1.2293 (19)	C9—H9C	0.9800
C1—C2	1.516 (2)	C10—C11	1.523 (2)
C1—H1B	0.9800	C10—C12	1.531 (2)
C1—H1C	0.9800	C10—H10A	1.0000
C1—H1D	0.9800	C11—H11A	0.9800
C2—C3	1.519 (2)	C11—H11B	0.9800
C2—C4	1.544 (2)	C11—H11C	0.9800
C2—H2B	1.0000	C13—C15	1.509 (3)
C3—H3A	0.9800	C13—C14	1.533 (3)
C3—H3B	0.9800	C13—C16	1.533 (3)
C3—H3C	0.9800	C14—H14A	0.9800
C5—C7	1.523 (2)	C14—H14B	0.9800
C5—C6	1.527 (2)	C14—H14C	0.9800
C5—C8	1.530 (2)	C15—H15A	0.9800
C6—H6A	0.9800	C15—H15B	0.9800
C6—H6B	0.9800	C15—H15C	0.9800
C6—H6C	0.9800	C16—H16B	0.9800
C7—H7A	0.9800	C16—H16C	0.9800
C7—H7B	0.9800	C16—H16A	0.9800
			
C4—N1—C5	126.13 (11)	C5—C8—H8C	109.5
C4—N1—H1A	116.9	H8A—C8—H8C	109.5
C5—N1—H1A	116.9	H8B—C8—H8C	109.5
C12—N2—C13	124.63 (13)	C10—C9—H9A	109.5
C12—N2—H2A	117.7	C10—C9—H9B	109.5
C13—N2—H2A	117.7	H9A—C9—H9B	109.5
C2—C1—H1B	109.5	C10—C9—H9C	109.5
C2—C1—H1C	109.5	H9A—C9—H9C	109.5
H1B—C1—H1C	109.5	H9B—C9—H9C	109.5
C2—C1—H1D	109.5	C9—C10—C11	110.23 (15)
H1B—C1—H1D	109.5	C9—C10—C12	109.85 (13)
H1C—C1—H1D	109.5	C11—C10—C12	108.96 (14)
C1—C2—C3	111.37 (16)	C9—C10—H10A	109.3
C1—C2—C4	109.28 (14)	C11—C10—H10A	109.3
C3—C2—C4	109.74 (14)	C12—C10—H10A	109.3
C1—C2—H2B	108.8	C10—C11—H11A	109.5
C3—C2—H2B	108.8	C10—C11—H11B	109.5
C4—C2—H2B	108.8	H11A—C11—H11B	109.5
C2—C3—H3A	109.5	C10—C11—H11C	109.5
C2—C3—H3B	109.5	H11A—C11—H11C	109.5
H3A—C3—H3B	109.5	H11B—C11—H11C	109.5
C2—C3—H3C	109.5	O2—C12—N2	123.37 (15)
H3A—C3—H3C	109.5	O2—C12—C10	120.91 (14)
H3B—C3—H3C	109.5	N2—C12—C10	115.72 (13)
O1—C4—N1	124.59 (14)	N2—C13—C15	110.67 (15)
O1—C4—C2	120.20 (14)	N2—C13—C14	110.00 (16)
N1—C4—C2	115.22 (12)	C15—C13—C14	111.19 (18)
N1—C5—C7	111.09 (12)	N2—C13—C16	105.46 (15)
N1—C5—C6	109.64 (12)	C15—C13—C16	110.0 (2)
C7—C5—C6	111.15 (14)	C14—C13—C16	109.32 (18)
N1—C5—C8	106.60 (12)	C13—C14—H14A	109.5
C7—C5—C8	108.50 (14)	C13—C14—H14B	109.5
C6—C5—C8	109.75 (13)	H14A—C14—H14B	109.5
C5—C6—H6A	109.5	C13—C14—H14C	109.5
C5—C6—H6B	109.5	H14A—C14—H14C	109.5
H6A—C6—H6B	109.5	H14B—C14—H14C	109.5
C5—C6—H6C	109.5	C13—C15—H15A	109.5
H6A—C6—H6C	109.5	C13—C15—H15B	109.5
H6B—C6—H6C	109.5	H15A—C15—H15B	109.5
C5—C7—H7A	109.5	C13—C15—H15C	109.5
C5—C7—H7B	109.5	H15A—C15—H15C	109.5
H7A—C7—H7B	109.5	H15B—C15—H15C	109.5
C5—C7—H7C	109.5	C13—C16—H16B	109.5
H7A—C7—H7C	109.5	C13—C16—H16C	109.5
H7B—C7—H7C	109.5	H16B—C16—H16C	109.5
C5—C8—H8A	109.5	C13—C16—H16A	109.5
C5—C8—H8B	109.5	H16B—C16—H16A	109.5
H8A—C8—H8B	109.5	H16C—C16—H16A	109.5
			
C5—N1—C4—O1	3.9 (3)	C13—N2—C12—O2	2.5 (3)
C5—N1—C4—C2	−175.58 (14)	C13—N2—C12—C10	−176.63 (15)
C1—C2—C4—O1	−63.0 (2)	C9—C10—C12—O2	49.6 (2)
C3—C2—C4—O1	59.4 (2)	C11—C10—C12—O2	−71.2 (2)
C1—C2—C4—N1	116.50 (17)	C9—C10—C12—N2	−131.26 (16)
C3—C2—C4—N1	−121.10 (17)	C11—C10—C12—N2	107.89 (17)
C4—N1—C5—C7	−60.1 (2)	C12—N2—C13—C15	59.7 (2)
C4—N1—C5—C6	63.11 (19)	C12—N2—C13—C14	−63.5 (2)
C4—N1—C5—C8	−178.15 (16)	C12—N2—C13—C16	178.7 (2)

### Hydrogen-bond geometry (Å, °)

**Table:**

*D*—H···*A*	*D*—H	H···*A*	*D*···*A*	*D*—H···*A*
N1—H1A···O2	0.88	2.03	2.8880 (16)	166
N2—H2A···O1^i^	0.88	2.10	2.9735 (16)	169

Symmetry codes:  (i) *x*, *y*, *z*+1.
