# *N*-*tert*-Butyl-2-methyl­propanamide. Corrigendum

**DOI:** 10.1107/S1600536811048902

**Published:** 2011-11-19

**Authors:** Kelly A. Kluge, Diana Fridlyand, Cora E. MacBeth, Kenneth I. Hardcastle

**Affiliations:** a Department of Chemistry, Emory University, 1515 Dickey Drive, Atlanta, GA 30322, USA

## Abstract

The name of one of the authors in the paper by Kluge *et al.* [*Acta Cryst.* (2011), E**67**, o2143] is corrected.

## Abstract 2

Corrigendum to *Acta Cryst.* (2011), E**67**, o2143.

In the paper by Kluge *et al.* (2011) ▶, the name of the second author is given incorrectly. The correct name is given above.
